# Comments on “Anatomically distinct fibroblast subsets determine skin autoimmune patterns”

**DOI:** 10.1097/CM9.0000000000002303

**Published:** 2022-08-10

**Authors:** Yudi Chen, Jianmin Chang

**Affiliations:** Department of Dermatology, Beijing Hospital, National Center of Gerontology, Institute of Geriatric Medicine, Chinese Academy of Medical Sciences, Beijing 100730, China.

Vitiligo is an autoimmune skin disorder characterized by white patches of the skin losing functional epidermal melanocytes. Progressive depigmentation of the skin impairs patients’ quality of life badly. More than 80% of patients with vitiligo have bilateral symmetric patterns of depigmentation, which is classified as nonsegmental vitiligo. However, few research revealed the cellular and molecular mechanisms that orchestrate the patterned activities of cutaneous immune cells in vitiligo patients. Understanding what orchestrates the activities of cutaneous immune cells is essential for determining the therapeutic targets of the disease.

Xu *et al*^[1]^ recently identified subsets of dermal fibroblasts that are responsible for driving patterned autoimmune activity, by using a robust mouse model of vitiligo that is based on the activation of endogenous auto-reactive CD8^+^ T cells that target epidermal melanocytes.^[1]^ Using advanced, powerful single-cell and imaging techniques, this study demonstrated the feedforward system between CD8^+^ T cells and skin fibroblasts which is of prime importance in the progression of vitiligo.^[1]^

Based on previous studies, CD8^+^ T cell infiltrating plays a direct role in melanocyte disappearance. Using a combination of single-cell analysis of skin samples from patients with vitiligo, cell-type-specific genetic knockouts, and engraftment experiment, the researchers identified that subsets of interferon (IFN) γ responsive fibroblasts are essential for skin recruitment, local aggregation, and cytotoxicity of CD8^+^ T cells targeting epidermal melanocytes. IFNγ-responsive fibroblasts mediate local aggregation of CD8^+^ T cells through the CXCL9/CXCL10–CXCR3 axis, which was further revealed by additional *in vitro* and *in vivo* experiments in the study.

The researchers also analyzed the clinical characteristics of 2265 nonsegmental vitiligo cases and found the frequency of vitiligo in different body regions varied. The back of the hand, the chest, and the back were the most susceptible to vitiligo, and the palm and arm the least. RNA-seq analysis of *in vitro* IFNγ-treated primary human dermal fibroblasts from different anatomic regions was conducted, revealing that the numbers and types of upregulated genes in response to IFNγ varied significantly among region-specific fibroblasts. Multiple IFNγ-induced chemokine genes (including *CXCL9*, *CXCL10*, and their human-specific variant *CXCL11*) were significantly upregulated in fibroblasts that were isolated from anatomic regions with a higher incidence of vitiligo, such as the back of the hand, the back of the foot, the chest, and the back. Using mouse models of vitiligo, this study further confirmed that CD8^+^ T cells preferentially aggregate toward a region with fibroblasts capable of stronger IFNγ responses, hence generating a patterned loss of melanocytes. Namely, regional distinct fibroblasts determine the autoimmune pattern of depigmentation in the skin.

This study leads to our better understanding of the functions of fibroblasts in vitiligo, as well as in other autoimmune diseases. Fibroblasts are mesenchymal cells that make up the stroma in organ tissues. They were traditionally considered as “immune neutral” cells, whose primary functions were thought to be the production, remodeling, and contraction of extracellular matrix. However, it is now clear that fibroblasts can serve as immune regulators across anatomical locations and diseases, highlighting their pivotal role in local tissue immunity. For example, in rheumatoid arthritis and inflammatory bowel disease, fibroblasts have been reported to lie at the center of positive immunity feedback loops.^[2]^

In terms of the pathogenesis of vitiligo, the result of this study is groundbreaking. This study sufficiently identified the positive-feedback loop between IFNγ-responsive fibroblasts and CD8^+^ T cells in vitiligo. Keratinocytes were thought to be predominantly responsible for T cell recruitment and they were considered to produce the bulk of chemokines.^[3,4]^ Few researchers pay attention to the pivotal role of fibroblasts in the pathogenesis of immunity in vitiligo. This study identified that IFNγ-responsive fibroblasts can recruit CD8^+^ T cells through *CXCL9* and *CXCL10* in vitiligo. The cyclical nature of the feedforward loop leads to the progressive aggregation of CD8^+^ T cells in areas with IFNγ-responsive fibroblasts resulting in the development of vitiligo.

More precisely, this study further revealed that in the pathogenesis of vitiligo, IFNγ-responsive dermal fibroblasts are not only uniquely required but also sufficient to induce local aggregation of CD8^+^ T cells. The researchers used a cell-type-specific knockout (KO) strategy to identify the irreplaceable role of fibroblasts in the pathogenesis of vitiligo. Compared to the KO of *Ifngr1* in endothelial cells, keratinocytes, myeloid immune cells, and melanocytes, only the mouse model that ablated *Ifngr1* in fibroblasts could not develop vitiligo under the melanoma-T_reg_-induced vitiligo model. On the other hand, intradermal injection of wild-type but not *Ifngr1* KO-fibroblasts into the tail skin of *Ifngr1*KO mice resulted in significant local aggregation of CD8^+^ T cells after the induction of vitiligo.

Skin fibroblasts display heterogeneity in different anatomical locations. The transcriptional signatures of the skin fibroblasts vary across the limbs, diverging from the torso toward the fingers or toes, suggesting that fibroblasts are imprinted with positional identity.^[4]^ Such imprinting occurs during development and is maintained in the postnatal period by epigenetic regulation of *HOX* genes.^[5]^ In this study, the regional variation of fibroblast responses to IFNγ was proven to be associated with the skin lesion distribution in nonsegmental vitiligo. Apart from vitiligo, many autoimmune skin disorders exhibit regional preference. The location-specific fibroblast subsets may contribute to the spatial distribution of skin lesions in other autoimmune skin diseases. The role of fibroblast-mediated immune response in other autoimmune skin disorders may need to be further explored.

Up to now, available treatments for vitiligo are limited, and therapeutical options mainly rely on drugs that target nonspecifically the inflammatory and immune responses such as topical and systemic steroids, and topical calcineurin inhibitors. The results of this study highlighted the essential role of IFNγ-responsive fibroblasts in the immune responses in vitiligo, which shed light on the potential role of fibroblast-targeting therapies. Firstly, blocking the activation signals may be a key approach to targeting fibroblasts. Although fibroblasts can respond to diverse cytokines and activation signals, this study has proven IFNγ to be one of the most significant ones. However, blocking IFNγ prevents fibroblast activation but is not specific; it may also block activation of many other cell types and reshape the disease response. Another approach to target inflammatory fibroblasts is to block the key cytokines and chemokines they secrete. This study has demonstrated that CXCL9 and CXCL10 are inflammatory fibroblast-derived factors critical for CD8^+^ T cell recruitment. Therefore, targeting these chemokine-chemokine receptor signaling and the downstream signaling proteins JAK1, JAK2, and STAT1 may also deserve further investigations. For example, case reports and open-label studies have recognized the therapeutic potential of JAK inhibitors in vitiligo.

This study may have the following limitations. Fibroblasts show heterogeneity even within a single organ. This study has not answered what specific features or unique expression profiles determined fibroblasts’ ability to respond to IFN-γ. We need to further explore the fibroblast cell subtypes that play a role in the pathogenesis of vitiligo. For example, we can perform gene ontology analysis on the transcriptome of fibroblast subtypes in mouse models and vitiligo patients to figure out the specific enrichment of IFN-γ pathway gene cell subsets. In the future, depleting vitiligo-specific pathogenic fibroblast subsets through targeting the potential distinct surface markers may serve as a possible treatment strategy. Besides, the expression patterns of fibroblasts may also have racial differences. The cellular cross-talk reported by this study may need to be further confirmed in different ethnic groups.

Generally speaking, fibroblasts are complex, multifaceted tissue-resident sentinel cells. Upon challenge with a range of different tissue insults, they help to initiate, govern, and moderate subsequent immune responses. However, in an autoimmune disease like vitiligo, inappropriate fibroblast activation facilitates disease progression by induction of pro-inflammatory properties. This study broadens our understanding of the cellular cross-talk between fibroblasts and T cells. Apart from skin, fibroblasts are ubiquitously present in essentially all organs of our body, the result of this study provides avenues for investigations of the potentially broad applications of this cellular cross-talk, which will enable the identification of targets for fibroblast therapeutics that may impact a range of inflammatory diseases.

## Conflicts of interest

None.
